# A pharmacodynamic study of rapid lactate metabolic modulation by intravenous L-arginine in brain metastases

**DOI:** 10.3389/fonc.2026.1845249

**Published:** 2026-05-28

**Authors:** Xueping Liu, Guobo Du, Nai Mu, Lang He

**Affiliations:** 1 Cancer Prevention and Treatment Institute of Chengdu, Department of Oncology, Chengdu Fifth People’s Hospital/The Second Clinical Medical College, Affiliated Fifth People’s Hospital of Chengdu University of Traditional Chinese Medicine, Chengdu, China; 2 Department of Medical Oncology, Affiliated Hospital of North Sichuan Medical College, Nanchong, Sichuan, China; 3 Geriatric Diseases Institute of Chengdu, Department of Orthopedics, Chengdu Fifth People’s Hospital/The Second Clinical Medical College, Affiliated Fifth People’s Hospital of Chengdu University of Traditional Chinese Medicine, Chengdu, China

**Keywords:** brain metastases, lactate, L-arginine, magnetic resonance spectroscopy, radiosensitization, radiotherapy, therapeutic window

## Abstract

**Objective:**

L-arginine may enhance radiotherapy efficacy through nitric oxide (NO)-mediated glycolysis inhibition; however, its real-time metabolic effects in brain metastases remain undefined. This proof-of-concept study aimed to characterize, for the first time *in vivo*, the rapid pharmacodynamic profile of intravenous L-arginine on lactate metabolism in brain metastases and generate hypotheses for future trials.

**Methods:**

Twenty patients with solid tumor brain metastases were randomized 1:1:1:1 to receive normal saline or 10 g, 20 g, or 30 g intravenous L-arginine. Serial magnetic resonance spectroscopy (MRS) was performed at baseline and multiple post-infusion timepoints (approximately 19–36 minutes) to quantify lactate dynamics. The primary endpoint was lactate reduction at T3 (approximately 30 minutes).

**Results:**

All patients completed the study. The pooled L-arginine group showed significantly greater lactate reduction at T3 compared with controls (median ΔLac_T3: −1.09 vs. 0.00, *p* = 0.0012). Lactate reduction was most prominent and consistent in the 30 g group, with a peak reduction of 63.5% at 30 minutes (ρ = −0.753, *p* < 0.001). No treatment-related adverse events were observed up to 24 hours post-infusion.

**Conclusion:**

This proof-of-concept study demonstrates that intravenous L-arginine rapidly and safely suppresses lactate metabolism in brain metastases, with a peak effect at approximately 30 minutes. The 30 g dose yielded the most robust metabolic suppression within the 10–30 g range. The “30 g L-arginine followed by radiotherapy within 30 minutes” regimen is proposed as a priority candidate for validation in future phase II trials. Clinical benefits require further confirmation in larger randomized controlled trials.

**Clinical Trial Registration:**

https://www.chictr.org.cn/, identifier ChiCTR2400080841.

## 1 Introduction

Brain metastases (BM) are a common complication of advanced solid tumors associated with poor prognosis. Radiotherapy is the cornerstone of local control for BM, and its efficacy is closely linked to metabolic reprogramming in the tumor microenvironment (1). Notably, abnormal glucose metabolism characterized by the Warburg effect and consequent lactate accumulation has been proven to be an important mechanism mediating radioresistance (2, 3). Consequently, targeting lactate metabolism has emerged as a promising strategy to enhance radiosensitivity.

L-arginine is a precursor for the synthesis of NO and plays an important role in tumor metabolism. Recently, Marullo et al. demonstrated that oral L-arginine significantly enhanced radiotherapy response in patients with BM, with mechanisms potentially related to NO-mediated inhibition of glycolysis and subsequent lactate reduction (4). However, limitations of oral routes such as substantial variable bioavailability (20–60%), individual differences, and potential gastrointestinal adverse reactions may reduce the stability and predictability of the therapy. In contrast, intravenous administration ensures rapid and complete systemic delivery, offering the potential for more reliable and controlled pharmacologic effects (5).

Despite the promising results of oral L-arginine, the real-time effects of intravenous L-arginine in human brain metastases remain undefined. The absence of key pharmacological parameters, such as the optimal dose, time to peak, and duration of action, hinders the design of precise combination strategies and clinical translation. Therefore, we conducted this prospective, randomized, proof-of-concept pharmacodynamic study to investigate, for the first time using serial *in vivo* MRS, the rapid and dynamic effects of intravenous L-arginine at three dose levels (10 g, 20 g, and 30 g) on lactate metabolism in patients with solid tumor BM (6). The primary objectives were to characterize the pattern of lactate reduction across doses and define the temporal window of peak metabolic effect. The results of this study will provide essential pharmacological guidance for the design of future phase II clinical trials evaluating the efficacy of intravenous L-arginine as a radiosensitizer for BM.

## 2 Methods

### 2.1 Trial design and patient eligibility criteria

**Figure 1:**
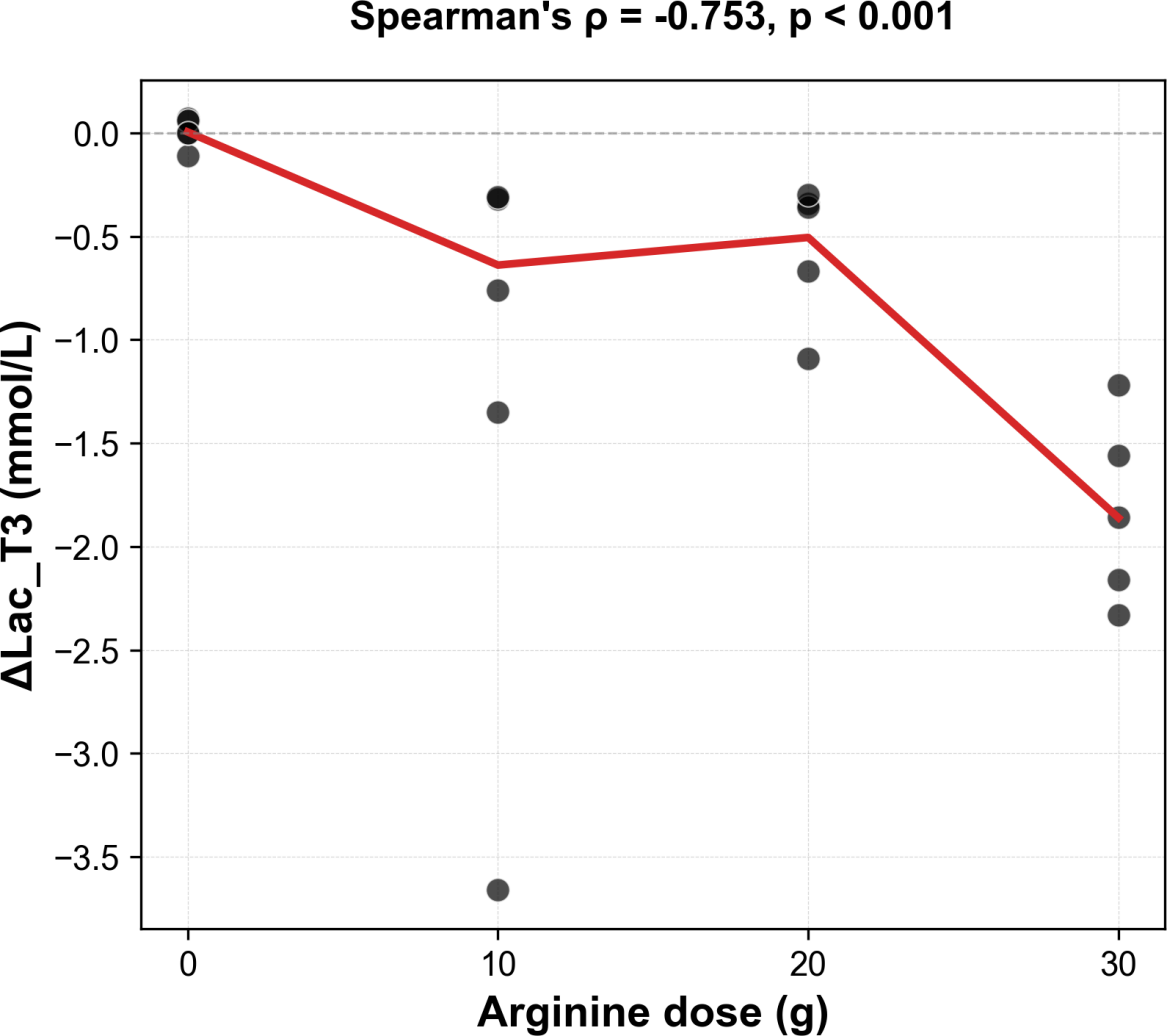
Study flow chart.

This study was designed as a prospective, single-center, open-label, randomized proof-of-concept pharmacodynamic trial (Approval No.:2023-017(科)-01). The flowchart of the technical route is shown in **Figure 1**. The protocol was approved by the Hospital Ethics Committee (Approval No.: 2023ER021-1), and written informed consent was obtained from all participants prior to enrollment. No formal sample size calculation was performed for this exploratory study. Based on previous similar metabolic pharmacodynamic studies, a sample size of 5 patients per group was selected to preliminarily evaluate the effect of intravenous L-arginine on lactate metabolism in brain metastases and generate hypotheses for future confirmatory trials. Due to the limited sample size, statistical results should be interpreted as indicative of trends rather than definitive conclusions, and no inferential claims about dose ranking can be made.

Inclusion criteria: (1) Age 18–75 years; (2) histologically or cytologically confirmed solid malignancy with at least one measurable brain metastasis deemed unsuitable for surgical resection by a multidisciplinary team; (3) target metastasis: diameter ≥ 1.0 cm, solid component > 50%, located in the cerebral hemisphere or cerebellum; (4) no prior radiotherapy to the brain and no systemic anticancer therapy (chemotherapy, targeted therapy, or immunotherapy) within 4 weeks; (5) Karnofsky Performance Status (KPS) score ≥ 70 and life expectancy > 3 months; (6) adequate liver and kidney functions.

Exclusion criteria: (1) Brain metastases unsuitable for MRS, including cystic or hemorrhagic lesions, or lesions with severe edema; (2) isolated leptomeningeal disease; (3) known contraindications or hypersensitivity to L-arginine or magnetic resonance imaging (MRI); (4) uncontrolled comorbidities, pregnancy, or lactation.

### 2.2 Intervention and randomization

Eligible patients were randomly assigned using a computer-generated sequence in a 1:1:1:1 ratio: a control group receiving an intravenous infusion of 250 mL 0.9% sodium chloride, or three treatment groups receiving intravenous L-arginine hydrochloride (Tianjin Jinyao Pharmaceutical, China; H12020999) at doses of 10 g, 20 g, or 30 g, each diluted in 250 mL 0.9% sodium chloride. All infusions were administered through a peripheral venous catheter at a constant rate (83 drops/min) as a single dose.

### 2.3 Multi-timepoint MRS protocol for lactate quantification

All scans were performed on a 3.0 Tesla MRI system (uMR 790, United Imaging Healthcare, Shanghai, China) (7). After routine clinical MRI sequences, a single-voxel point-resolved spectroscopy (PRESS) sequence was employed for MRS data acquisition (8). Spectra were acquired at five time points: baseline (T0, pre-infusion) and four consecutive windows post-infusion: T1 (19.00–23.35 min), T2 (23.35–27.70 min), T3 (27.70–32.05 min), and T4 (32.05–36.4 min). The voxel was precisely placed on the solid enhancing component of the target metastasis, avoiding cystic, necrotic, or edematous regions.

Lactate levels were quantified using LCModel software (Version 6.3-1L, Stephen Provencher, Oakville, ON, Canada) and expressed as the ratio of lactate peak area to creatine (Cr) peak area. Quality control was performed using signal-to-noise ratio (SNR) and Cramér-Rao lower bound (CRLB). A CRLB < 20% was considered acceptable for quantitative analysis. All spectra were visually inspected by two independent board-certified radiologists to exclude voxels with severe lipid contamination or motion artifacts. No spectra were excluded due to poor quality. Detailed quality control parameters are provided in **Supplementary Table 1**.

### 2.4 Study endpoints

The primary pharmacodynamic endpoint was the change in tumor lactate level from baseline at T3 (ΔLac_T3). Secondary endpoints were: (1) Percentage change in lactate at each time point. (2) Pattern of lactate reduction across different doses. (3) Kinetics of lactate change over time. (4) Safety, with all adverse events recorded.

### 2.5 Statistical analysis

Given the exploratory nature and limited sample size, non-parametric statistical methods were primarily employed. The Mann–Whitney *U* test was used to compare ΔLac_T3 between: 1) each L-arginine group versus the control group, 2) the pooled L-arginine groups (10–30 g) versus the control group. Spearman’s rank correlation was used to evaluate the association between L-arginine dose and ΔLac_T3. Changes in lactate levels over time (T0–T4) are presented descriptively for each group.

Formal statistical comparisons between the three L-arginine dose groups were not performed due to significant baseline imbalance in lactate levels and the floor effect in the 20 g group. All statistical analyses were conducted using GraphPad Prism 9.0 and SPSS 29.0. A two-sided *p* < 0.05 was considered statistically significant.

## 3 Results

### 3.1 Patient characteristics

This study included a total of 20 eligible patients, all of whom underwent randomization and completed all study procedures. The baseline demographic and clinical characteristics of the four groups are summarized in **Table 1**. Although stratified randomization was not performed due to the exploratory nature of the study, the groups were generally balanced and comparable in key indicators, such as age, sex, primary tumor type, target lesion characteristics, and KPS score. Notably, intergroup heterogeneity existed in baseline lactate levels: the 20 g group had substantially lower baseline lactate, creating a floor effect that limits valid direct comparisons of absolute reduction across doses.

**Table 1:** Baseline demographics and clinical characteristics.

Characteristic	Control (n = 5)	10 g Arg (n = 5)	20 g Arg (n = 5)	30 g Arg (n = 5)
Age, years – median (range)	62 (58–71)	61 (54–68)	57 (52–60)	59 (55–65)
Gender – male, n (%)	4 (80%)	3 (60%)	4 (80%)	3 (60%)
Primary Tumor Type, n (%)				
Lung Adenocarcinoma	2 (40%)	4 (80%)	5 (100%)	1 (20%)
Lung Squamous Cell Carcinoma	0	1 (20%)	0	2 (40%)
Small Cell Lung Cancer	2 (40%)	0	0	1 (20%)
Other	1 (20%)	0	0	1 (20%)
Number of Brain Metastases – multiple, n (%)	4 (80%)	3 (60%)	3 (60%)	3 (60%)
Target Lesion Size – ≥ 3 cm, n (%)	0	1 (20%)	2 (40%)	2 (40%)
Baseline Lactate (T0) – median (range)	3.43 (2.42–4.96)	3.47 (1.99–4.83)	1.39 (0.81–1.93)	3.13 (2.25–4.81)

### 3.2 Rapid lactate suppression following intravenous l-arginine infusion

**Figure 2:**
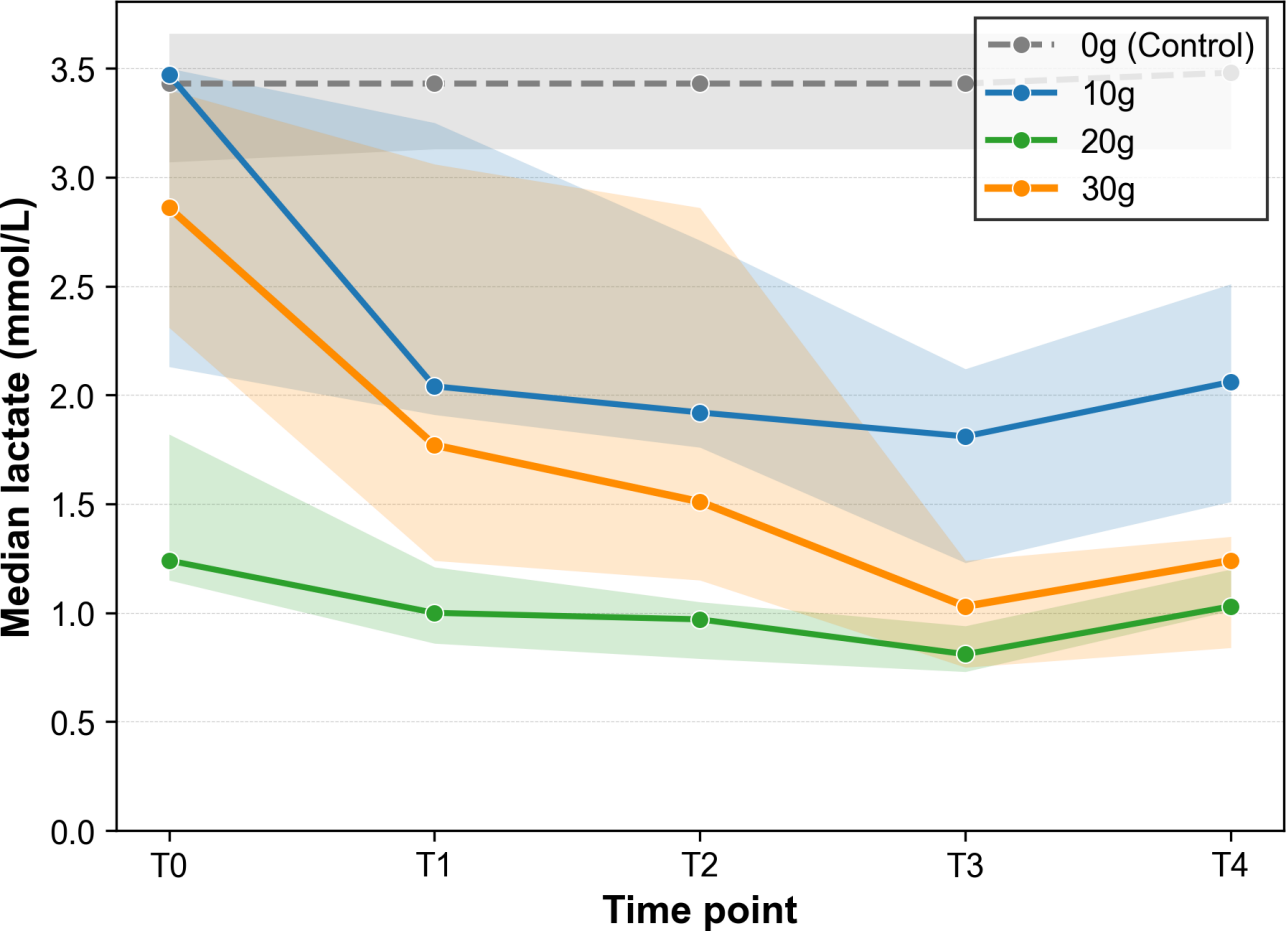
Comparison of lactate reduction between control and combined l-arginine groups at T3.

To determine whether intravenous L-arginine effectively reduces tumor lactate, we compared ΔLac_T3 at T3 between the pooled L-arginine groups (10–30 g) and the control group. As shown in **Figure 2**, lactate reduction was significantly greater in the L-arginine group. Median ΔLac_T3 was -1.09 in the pooled treatment group versus 0.00 in the control group. This difference was significant (*p* = 0.0012). This result confirms for the first time *in vivo* that intravenous L-arginine can rapidly and significantly inhibit lactate metabolism in brain metastases.

### 3.3 Lactate reduction across different dose groups

**Figure 3:**
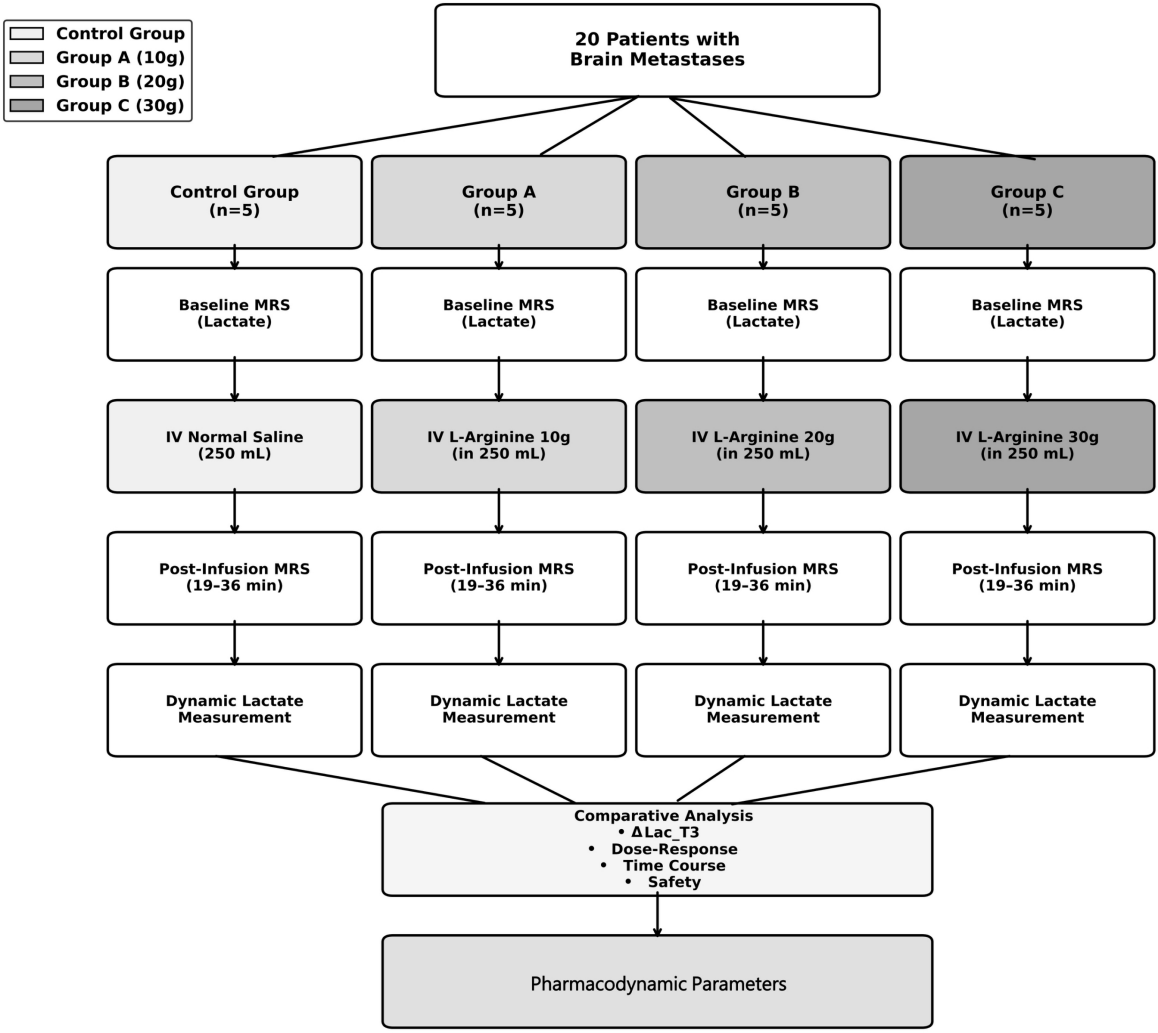
Pattern of lactate reduction across different l−arginine dose groups at T3.

We next evaluated the trend between lactate reduction and L-arginine dose. Spearman’s rank correlation revealed a strong negative correlation (ρ=-0.753, *p* < 0.001), indicating a trend toward greater lactate reduction with higher doses. This pattern of lactate reduction across doses is illustrated in **Figure 3**.

### 3.4 Pharmacodynamic time course and descriptive findings

**Figure 4:**
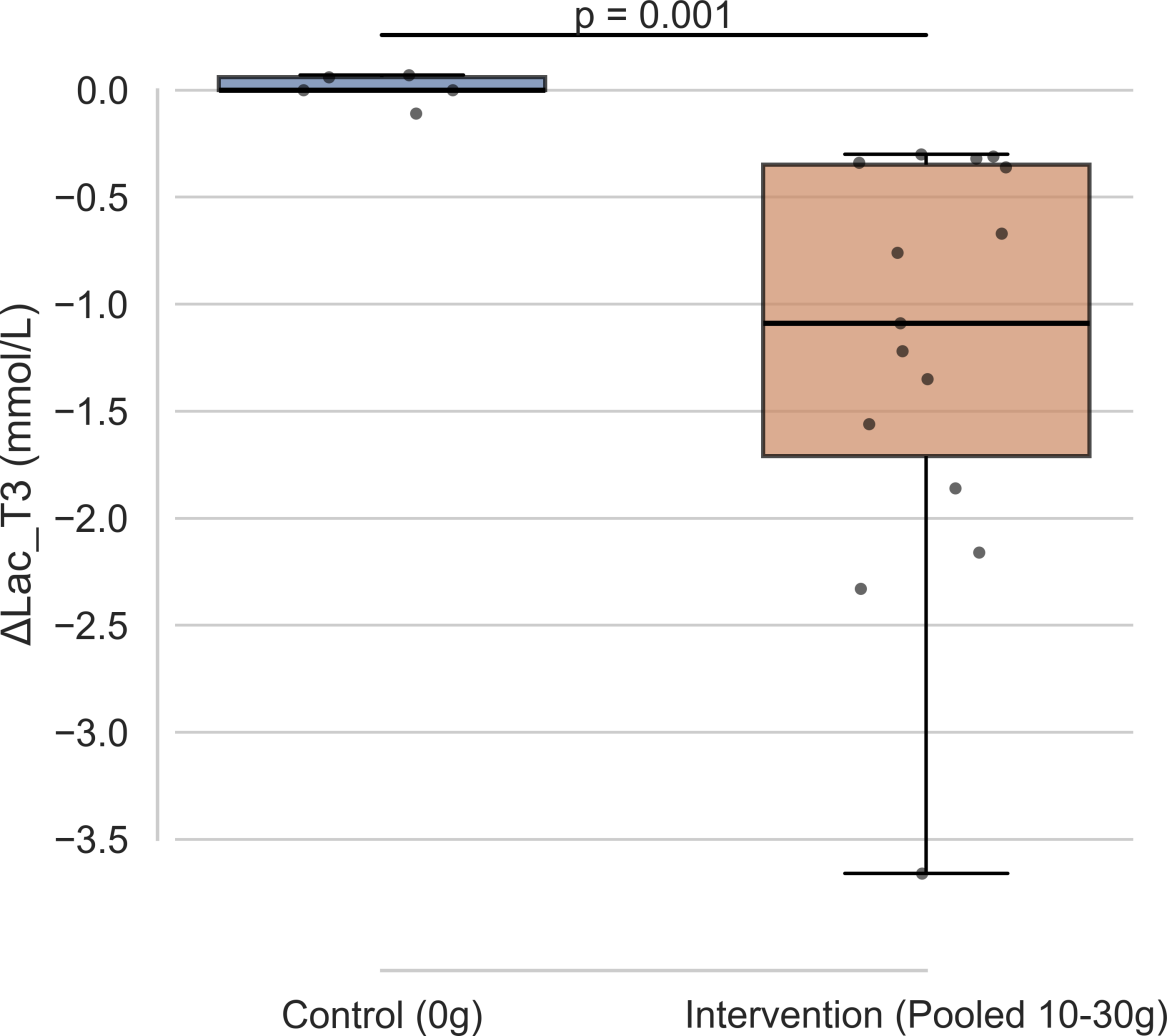
Pharmacodynamic time course of different dose groups.

To illustrate how lactate levels changed over time, we measured them at five time points: baseline (T0) and four consecutive windows after infusion (T1–T4). **Figure 4** presents the median lactate levels for each dose group. All L-arginine-treated groups showed a consistent pattern: lactate began to decrease at T1, reached its lowest point at T3 (around 30 min after infusion), and remained low through T4.

Median ΔLac_T3 values were -0.76, -0.36, and -1.86 in the 10 g, 20 g, and 30 g L-arginine groups, with median reductions of 38.2%, 34.7%, and 63.5%, respectively. All three L-arginine groups showed significantly greater lactate reduction than the control group (all *p* = 0.0119). The pooled treatment group confirmed a robust effect versus control (*p* = 0.0012) (**Table 2**). The 30 g group achieved the largest lactate reduction (63.5%).

**Table 2:** Summary of pharmacodynamic endpoints.

Group	ΔLac at T3, median (IQR)	% change at T3, median (IQR)	vs. control (Mann–Whitney *U* Test)
Control (0 g)	0.00 (0.00, 0.06)	0.0% (0.0%, 1.4%)	—
10 g L-Arg	-0.76 (-1.35, -0.32)	-38.2% (-38.9%, -15.0%)	*U* = 0.00, *p* = 0.0119, *r* = -0.826
20 g L-Arg	-0.36 (-0.67, -0.34)	-34.7% (-44.4%, -29.6%)	*U* = 0.00, *p* = 0.0119, *r* = -0.826
30 g L-Arg	-1.86 (-2.16, -1.56)	-63.5% (-67.5%, -54.2%)	*U* = 0.00, *p* = 0.0119, *r* = -0.826
Treatment (10–30 g)	-1.09 (-1.71, -0.35)	-38.9% (-61.7%, -32.1%)	*U* = 0.00, *p* = 0.0012, *r* = -0.736

Direct head-to-head statistical comparisons between different L-arginine dose groups were not performed due to significant baseline imbalance in lactate levels (floor effect in the 20 g group). Comparisons are only presented versus the control group.

Direct head-to-head statistical comparisons between L-arginine dose groups were not performed due to significant baseline lactate imbalance and a marked floor effect in the 20 g group. The data are presented descriptively, and the 30 g group is highlighted as showing the most consistent and largest effect.

### 3.5 Tolerability and adverse event profile

All 20 patients completed infusions and serial MRS scans. Safety was monitored continuously during infusion and for 24 hours post-infusion. L-arginine was well tolerated at all doses (10–30 g). No treatment-related adverse events were observed, including hypotension, headache, nausea, vomiting, or increased intracranial pressure. Vital signs remained stable throughout the observation period.

## 4 Discussion

### 4.1 Principal findings

Increasing evidence indicates that, to meet the demands of rapid proliferation and adapt to the harsh microenvironment, the L-arginine metabolic pathway of tumor cells undergoes significant reprogramming, making it an attractive target for cancer therapy (3, 9, 10). As a proof-of-concept exploratory study, this investigation using real-time *in vivo* MRS provides the first systematic characterization of the rapid metabolic effects of intravenous L-arginine on lactate in brain metastases. Principal findings: (i) Intravenous L-arginine can rapidly and significantly reduce lactate levels in brain metastases, with the peak effect occurring approximately 30 minutes after infusion; (ii) Lactate reduction was most prominent in the 30 g group; (iii) Single-dose safety and tolerability up to 24 hours are favorable across the 10–30 g range. These findings support the rapid operability of the L-arginine–NO–glycolysis axis in humans and provide preliminary pharmacodynamic parameters for future confirmatory trials.

### 4.2 L-arginine in radiation oncology

Early research on L-arginine in radiation oncology focused on nutritional support and radiation injury repair (11, 12), such as reducing radiation-induced mucositis (13). Following the elucidation of NO signaling, research increasingly centered on the L-arginine-NO axis for radiosensitization (14, 15). With in-depth study of tumor metabolic reprogramming (16, 17), two seemingly different but theoretically based strategies have gradually emerged.

One is L-arginine deprivation sensitization therapy, which uses L-arginine decarboxylase and L-arginine deiminase to deplete L-arginine in the tumor microenvironment to exert metabolic stress and aggravate radiation damage, mainly for L-arginine-auxotrophic tumors, such as head and neck squamous cell carcinoma, glioblastoma, and pancreatic cancer with low expression of argininosuccinate synthetase 1 (ASS1) (18–20). A recent study has shown that L-arginine deprivation therapy combined with radiotherapy was also effective for non-L-arginine-auxotrophic glioblastoma tumors expressing ASS1 (21).

Another strategy is arginine supplementation therapy, which uses exogenous high-dose L-arginine supplementation to drive robust NO production and enhance radiation sensitivity. The pivotal 2021 clinical trial by Marullo et al. provided key preclinical and early clinical evidence for this strategy (4). In their clinical study, approximately 78% of patients receiving L-arginine treatment experienced complete or partial remission of brain tumors, compared with only 22% in the control group, demonstrating the promising efficacy of oral L-arginine combined with whole-brain radiotherapy in patients with brain metastases.

However, oral administration is limited by highly variable bioavailability due to first-pass metabolism, gastrointestinal adverse effects, and inability to achieve predictable peak concentrations for precise timing with radiotherapy (5). Our study addresses these limitations by using intravenous L-arginine, which provides 100% bioavailability and precise temporal control. For the first time, we provide real-time, quantitative *in vivo* evidence of L-arginine-mediated lactate suppression in human brain metastases using serial magnetic resonance spectroscopy. The rapid lactate decline, temporally aligned with expected peak plasma L-arginine levels, directly supports the proposed metabolic reprogramming mechanism.

### 4.3 Mechanistic insights: the l-arginine-NO-glycolysis axis and beyond

**Figure 5:**
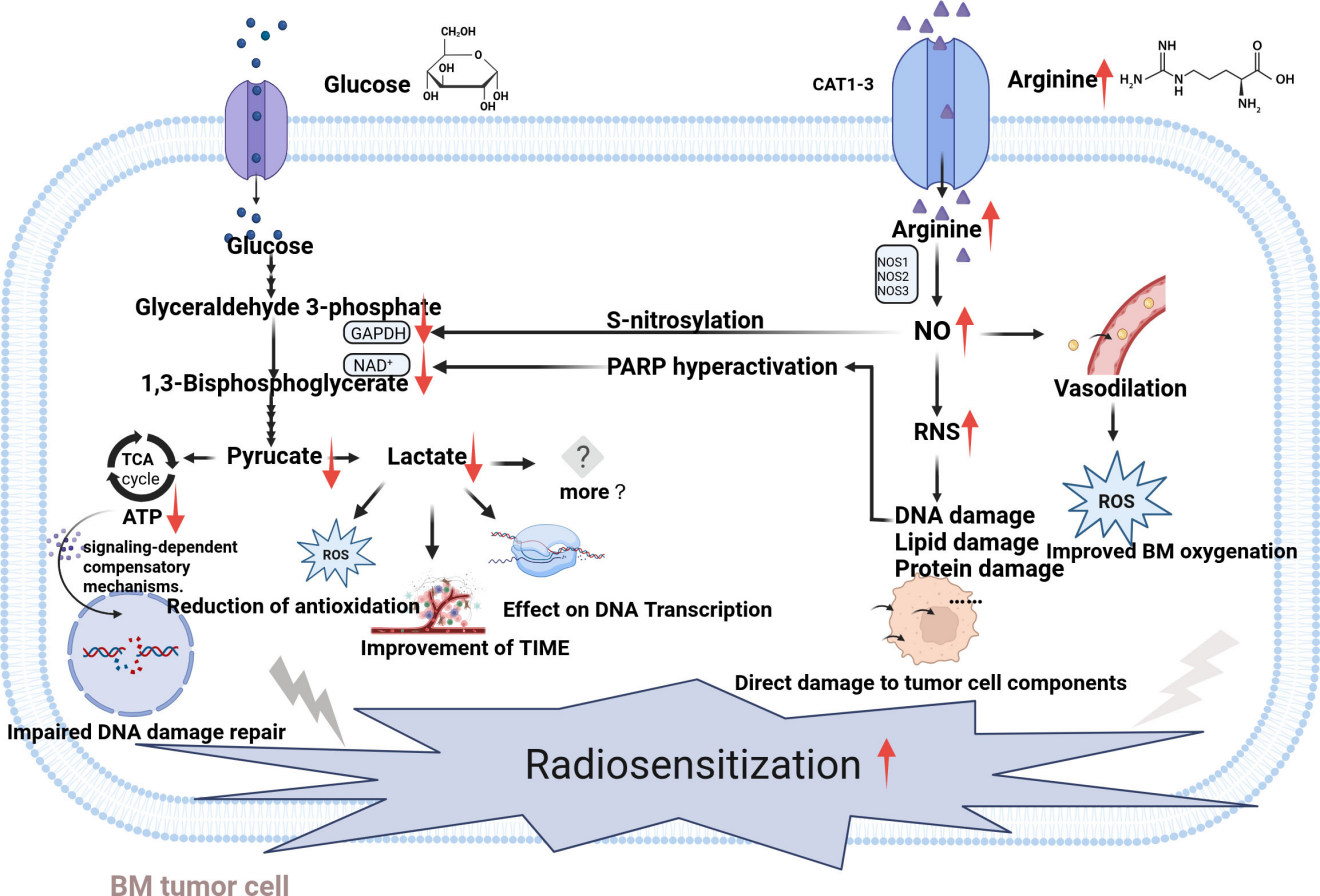
Mechanism diagram of l-arginine-mediated radiosensitization.

The main molecular mechanism of L-arginine radiosensitization is illustrated in **Figure 5**. Exogenous L-arginine enters tumor cells through cationic amino acid transporters (CATs) and drives inducible nitric oxide synthase (iNOS) to produce a large amount of NO (22). High concentrations of NO inhibit glyceraldehyde-3-phosphate dehydrogenase (GAPDH), a key rate-limiting enzyme in glycolysis, through S-nitrosylation (23). The inactivation of GAPDH directly blocks glycolysis and further reduces the production of lactic acid, which is exactly the pharmacological basis of the rapid decline of lactate we observed on MRS (24, 25).

ATP and NAD+ in tumor cells, meanwhile, decrease sharply. NAD+ is the only substrate of poly ADP-ribose polymerase (PARP), a critical enzyme in DNA damage repair. NAD+ depletion impairs PARP activation after radiation-induced DNA damage, which eventually prevents tumor cells from effectively repairing sublethal radiation damage, thereby promoting cell death (4, 26, 27).

Notably, lactate is not merely a glycolytic byproduct but functions as a critical signaling molecule linking tumor metabolism, radioresistance, and immunosuppression (28). A lactate-rich microenvironment promotes radioresistance through multiple mechanisms: it directly scavenges radiation-induced reactive oxygen species (ROS), upregulates DNA damage repair pathways, and activates pro-survival signaling cascades (25). Lactate-driven extracellular acidification further impairs effector T cell and natural killer cell function, polarizes macrophages toward an immunosuppressive M2 phenotype, and blunts the immunogenic cell death induced by radiotherapy (29–33).

Recent advances have revealed that lactate also mediates post-translational modification through protein lactylation, a novel epigenetic mechanism that regulates gene expression and promotes tumor stemness and therapy resistance (34). This finding expands our understanding of lactate’s oncogenic roles beyond metabolism and provides an additional mechanistic basis for the potential radiosensitizing effect of L-arginine-induced lactate reduction.

Therefore, the rapid lactate reduction observed in our study has broader clinical implications: (1) Lactate can serve as a real-time pharmacodynamic biomarker for L-arginine treatment, enabling individualized dose titration based on early metabolic response. (2) L-arginine-induced lactate depletion may not only directly enhance radiosensitivity but also remodel the immunosuppressive tumor microenvironment, providing a strong rationale for exploring triple combinations of radiotherapy, L-arginine, and immune checkpoint inhibitors. Importantly, these implications are based on a surrogate biomarker and require validation with clinical endpoints.

### 4.4 Clinical translational implications: pharmacodynamic parameters guiding trial design

As an exploratory proof-of-concept study, the core pharmacodynamic parameters we defined have direct clinical translational implications for L-arginine combined with radiotherapy.

First, the lactate nadir occurred at around 30 minutes after intravenous L-arginine infusion, which is highly consistent with the known pharmacokinetic peak of intravenous L-arginine (5). This finding clearly defines the optimal “window of opportunity” for radiotherapy, when tumor glycolytic metabolism is maximally suppressed.

Second, Spearman correlation analysis showed that lactate reduction became more pronounced with increasing L-arginine dose within the safe 10–30 g range, with the 30 g group achieving the largest median lactate reduction of 63.5% at T3. Notably, formal head-to-head statistical comparisons between different L-arginine dose groups were not performed due to significant baseline lactate imbalance and a floor effect in the 20 g group. Based on these findings, we propose the “30 g intravenous L-arginine followed by radiotherapy within 30 minutes” regimen as the priority candidate regimen for hypothesis validation in future phase II trials, rather than a definitively optimized strategy.

While single-dose safety up to 24 hours was favorable in this study, rigorous clinical monitoring is required when translating to multi-dose fractionated radiotherapy regimens. Particular attention should be paid to potential NO-mediated vasodilatory effects, including transient hypotension, headache, and intracranial pressure changes in patients with brain metastases (35).

In addition, the serial MRS technique used in this study enables non-invasive, real-time monitoring of intratumoral lactate dynamics (4, 36, 37). Integrating this method into routine radiotherapy workflows may allow real-time tracking of tumor metabolic responses, supporting adaptive and personalized treatment adjustments.

### 4.5 Limitations and future directions

Several critical limitations of this proof-of-concept study must be explicitly acknowledged:

1. Small sample size and lack of stratified randomization: This resulted in significant baseline heterogeneity in lactate levels, particularly a marked floor effect in the 20 g group, which precludes valid head-to-head comparisons between different L-arginine dose groups. Therefore, the primary contribution of this study is to demonstrate the existence of the rapid metabolic effect, the pattern of progressively greater lactate reduction with higher doses, and its temporal profile, rather than to establish a definitive dose ranking.
2. Lack of direct evidence for target engagement: We did not measure plasma arginine, tumoral NOS2, or NO metabolites; alternative mechanisms (e.g., altered lactate clearance due to blood flow changes) cannot be excluded (4, 38, 39).
3. Surrogate endpoint only: No clinical efficacy data are presented. The correlation of lactate reduction with objective response rate (ORR), intracranial progression-free survival (iPFS), overall survival (OS), or other clinical hard endpoints requires validation in randomized trials.
4. Limited safety follow-up: Safety was only monitored for 24 hours after a single dose. The long-term safety and tolerability of repeated L-arginine administration in the setting of fractionated radiotherapy have not been evaluated in this study.

Based on these limitations, we propose the following future directions:

1. A randomized phase II trial with ORR, iPFS, and OS as primary endpoints to validate the clinical benefit of the “30 g–30 min” regimen, with stratification by baseline lactate and ASS1/NOS2 expression;
2. Incorporation of plasma arginine/NO metabolite measurements to establish a complete pharmacodynamic evidence chain;
3. Exploration of synergy with immune checkpoint inhibitors and improved targeted delivery systems (e.g., liposomes, nanoparticles) (40–44).

## 5 Conclusion

This proof-of-concept study demonstrates that intravenous infusion of L-arginine can rapidly and safely inhibit lactate metabolism in brain metastases, with peak effect occurring approximately 30 minutes after infusion. The 30 g dose showed the most pronounced metabolic suppression. These findings provide critical pharmacodynamic guidance for the design of future clinical trials evaluating L-arginine as a metabolic radiosensitizer for brain metastases. While the clinical benefit of this approach remains to be established, the favorable safety profile and widespread availability of L-arginine make it a promising candidate for further investigation.

## Data Availability

The raw data supporting the conclusions of this article will be made available by the authors, without undue reservation.
