# Supplementary material for: A pharmacodynamic study of rapid lactate metabolic modulation by intravenous L-arginine in brain metastases
Source: Front Oncol. 2026 May 28;16:1845249. doi: 10.3389/fonc.2026.1845249 (PMC13253284; doi:10.3389/fonc.2026.1845249)
Supplement: Supplementary file 1 [file DataSheet1.pdf]

Supplementary Table S1. MRS Quality Control Parameters for Lactate Quantification

Group	Time Point	SNR, median (range)	CRLB (%), median (range)
Control (0 g)	T0	12.5 (8.2–18.3)	14.2 (9.5–19.8)
	T1	11.8 (7.9–17.6)	15.1 (10.2–20.5)
	T2	12.1 (8.0–18.0)	14.8 (9.8–19.9)
	T3	12.3 (8.1–18.2)	14.5 (9.6–20.1)
	T4	11.9 (7.8–17.9)	15.3 (10.0–20.8)
10 g L-Arg	T0	13.2 (9.0–19.5)	13.5 (8.5–18.9)
	T1	12.8 (8.5–18.8)	14.2 (9.0–19.5)
	T2	12.5 (8.3–18.3)	14.8 (9.2–20.0)
	T3	11.9 (8.0–17.5)	15.5 (9.8–21.2)
	T4	12.0 (8.1–17.8)	15.1 (9.5–20.5)
20 g L-Arg	T0	14.1 (9.8–21.2)	16.1 (11.8–21.5)
	T1	13.5 (9.2–20.1)	15.2 (10.1–19.9)
	T2	13.0 (8.8–19.5)	14.9 (9.6–19.6)
	T3	12.2 (8.3–18.0)	15.2 (9.0–20.8)
	T4	12.5 (8.5–18.5)	14.8 (8.8–19.8)
30 g L-Arg	T0	13.8 (9.5–20.5)	13.0 (8.0–18.0)
	T1	13.0 (8.8–19.2)	13.8 (8.5–19.0)
	T2	12.5 (8.3–18.3)	14.5 (9.0–19.8)
	T3	11.5 (7.8–17.0)	16.0 (10.0–22.0)
	T4	11.8 (8.0–17.5)	15.5 (9.5–21.0)
